# Supplementary material for: Revisiting the Plasmodium sporozoite inoculum and elucidating the efficiency with which malaria parasites progress through the mosquito
Source: Nat Commun. 2024 Jan 25;15:748. doi: 10.1038/s41467-024-44962-4 (PMC10811227; doi:10.1038/s41467-024-44962-4)
Supplement: Supplementary file 1 — Supplementary information [file 41467_2024_44962_MOESM1_ESM.pdf]

Supplementary Information for

Revisiting the *Plasmodium* sporozoite inoculum and elucidating the efficiency with which malaria parasites progress through the mosquito

Sachie Kanatani[1,2§]*, Deborah Stiffler[1,2§], Teun Bousema[3], Gayane Yenokyan[4], Photini Sinnis[1,2]*

[1]Department of Molecular Microbiology & Immunology, Johns Hopkins Bloomberg School of Public Health, Baltimore MD
[2]Johns Hopkins Malaria Institute, Johns Hopkins Bloomberg School of Public Health, Baltimore MD
[3]Department of Medical Microbiology & Radboud Center for Infectious Diseases, Radboud University Medical Center, Nijmegen, the Netherlands
[4]Department of Biostatistics, Johns Hopkins Bloomberg School of Public Health, Baltimore, MD

[§]contributed equally
*corresponding authors:
Sachie Kanatani skanata1@jhu.edu
Photini Sinnis psinnis1@jhu.edu phone: 410 502 6918

Supplementary Figures S1 to S5

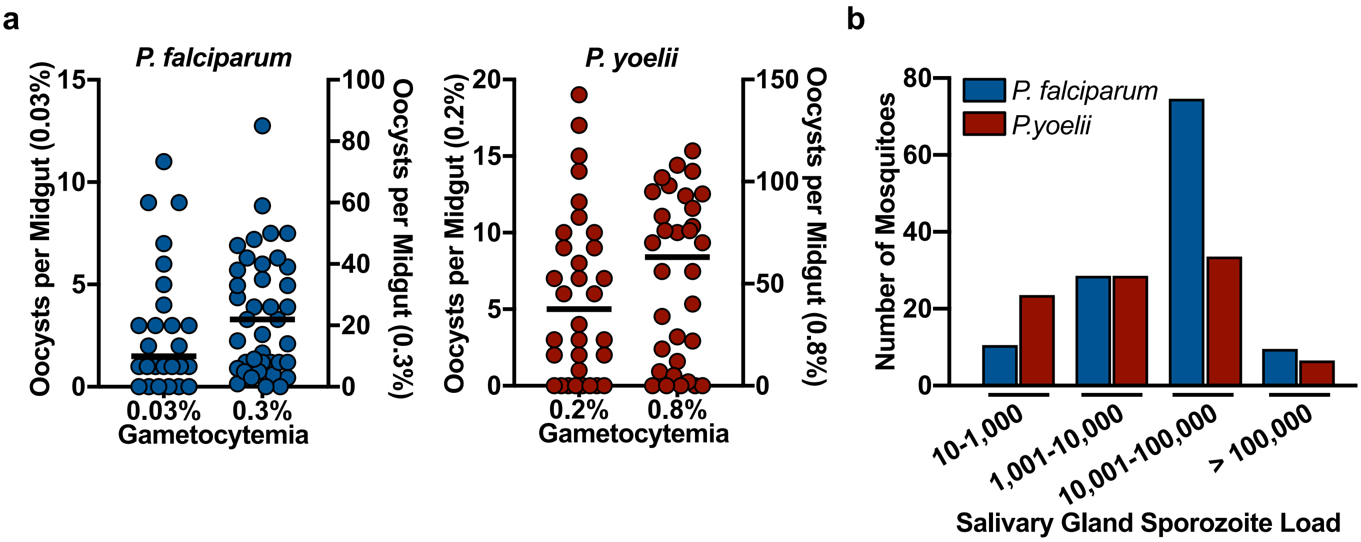

Supplementary Fig. 1 Generation of mosquitoes with a range of infection intensities. a *Anopheles stephensi* mosquitoes were fed on gametocyte cultures (*P. falciparum*) or mice (*P. yoelii*) with the indicated gametocytemias. On day 10 (*P. falciparum*) or 7 (*P. yoelii*) post-infection, mosquito midguts were harvested, and oocysts were counted. Each dot represents the number of oocysts from a single mosquito midgut, with bars indicating the median. Data were pooled from two independent mosquito cycles (*P. falciparum*: total n = 65, *P. yoelii*: n = 64). **b** Salivary gland sporozoite load of *P. falciparum* (blue) and *P. yoelii* (red) infected mosquitoes used in Figure 1 (*P. falciparum*) and Figure 4 (*P. yoelii*).

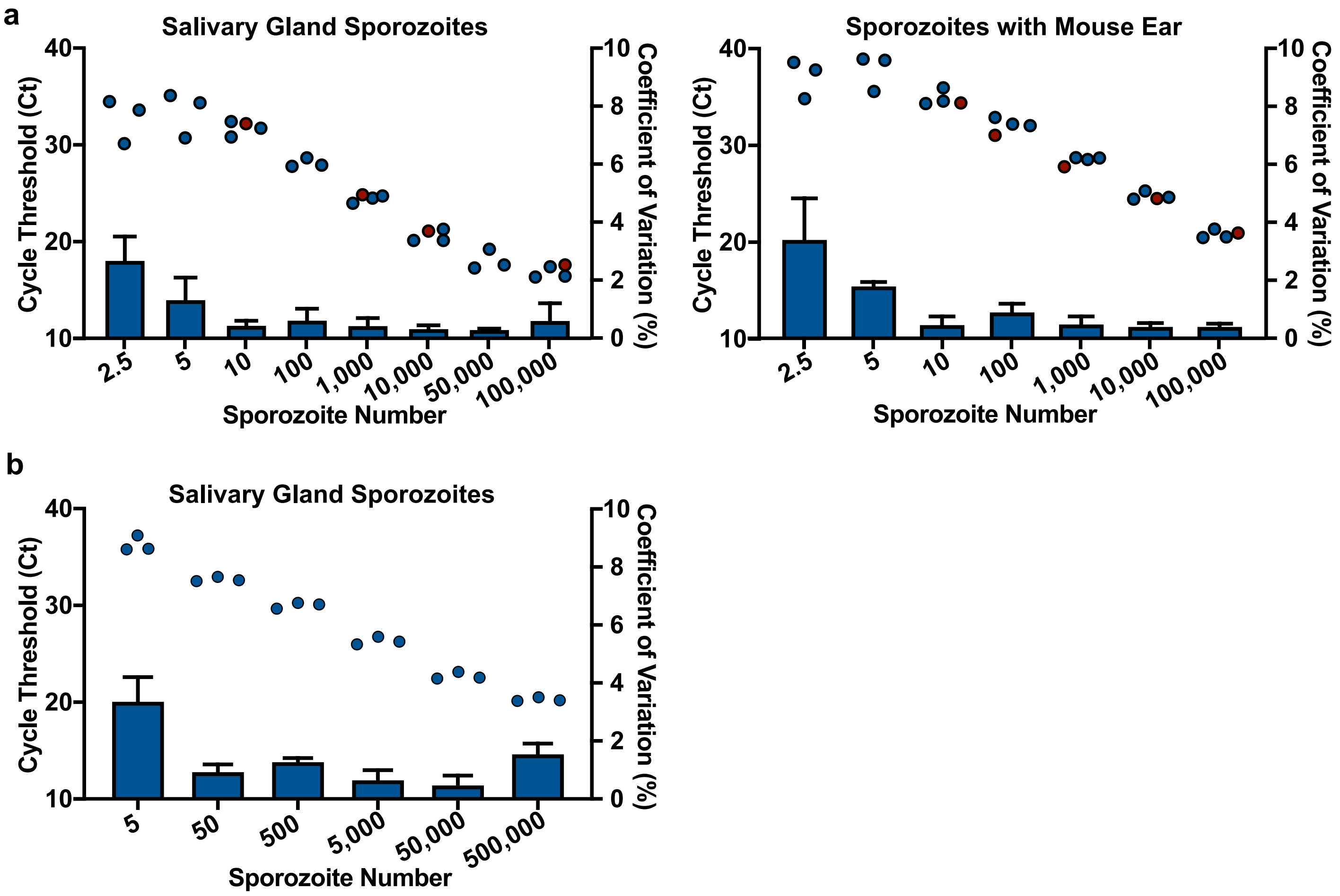

Supplementary Fig. 2 qPCR standard curves used to quantify sporozoites. Genomic DNA was isolated from serial dilutions of *P. falciparum* sporozoites, with or without ear tissue as indicated, and qPCR was performed with either LSUE **a** or 18S rRNA **b** primers. Dots indicate the mean cycle threshold of technical triplicates (left y-axis) and bars indicate the coefficient of variation (+/- standard deviation) of these samples (right y-axis). The blue dots indicate *P. falciparum* sporozoites and the red dots indicate *P. yoelii* sporozoites. The limit of detection (LOD) was set at 95% sample positivity; with LSUE primers this was 2.5 sporozoites. The limit of quantification (LOQ) was set at 100% sample positivity with a coefficient of variation less than 2%; with LSUE primers this was 10 sporozoites.

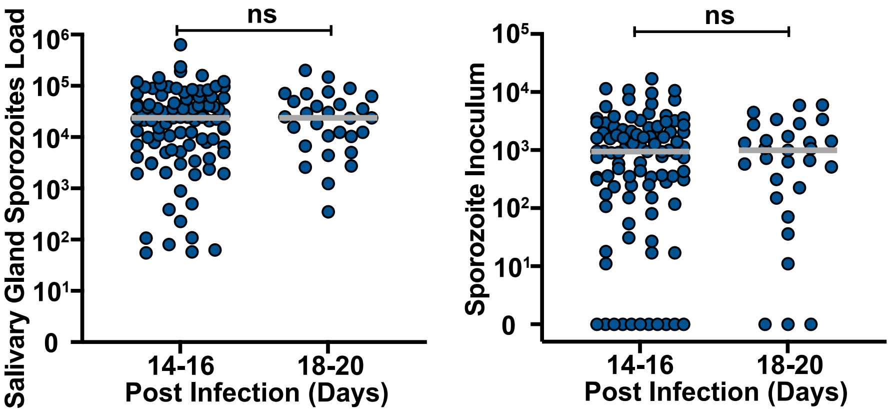

Supplementary Fig. 3 *Plasmodium falciparum* infected mosquitoes have comparable salivary gland sporozoite loads and inoculate similar quantities of sporozoites on days 14-16 and 18-20 post infection. The salivary gland sporozoite load (left graph) and sporozoite inoculum (right graph) and are not significantly different on 14-16 versus 18-20 post infection (ns, not significant, P > 0.5 [two-tailed Mann Whitney test]). Each dot represents a single mosquito, and bars indicate the median

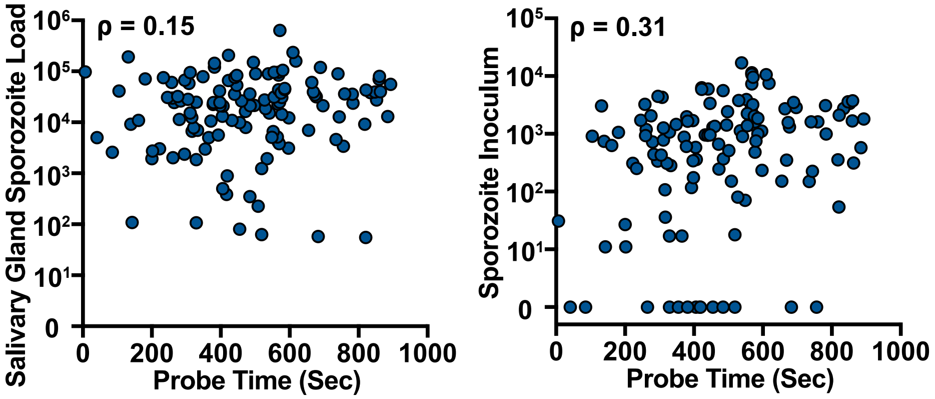

Supplementary Fig. 4 Duration of mosquito probing is not impacted by salivary gland sporozoite load and does not correlate with sporozoite inoculum size. The duration of mosquito probing was recorded for all mosquitoes used in the inoculum experiments. We found no correlation between mosquito salivary gland load and probe time (left graph, Spearman correlation ρ = 0.15; P = 0.085) nor between inoculum size and probe time (right graph, Spearman correlation ρ = 0.31; P = 0.0004).

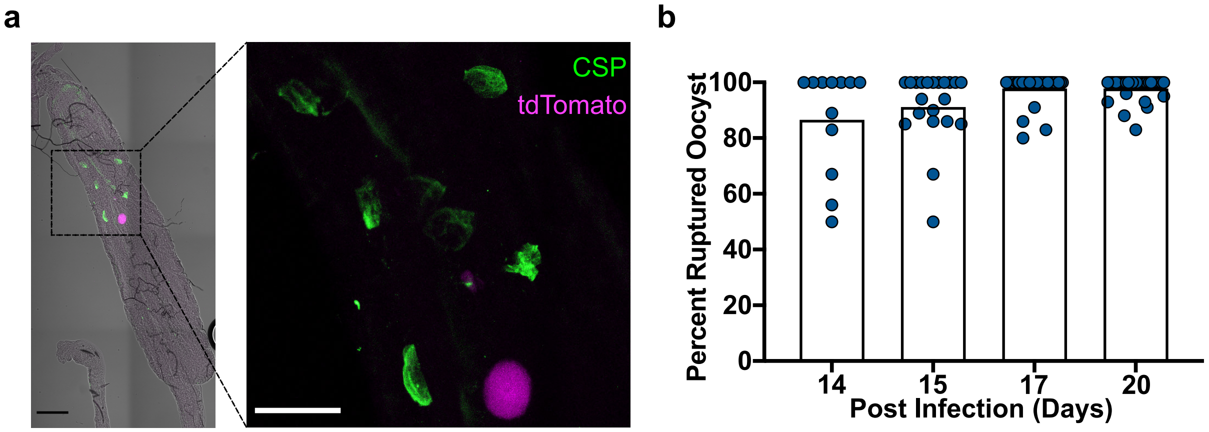

Supplementary Fig. 5 Utilizing CSP-staining to quantify the number of ruptured oocysts.
a Representative image of mosquito midgut infected with tdTomato-expressing *P. falciparum*. The midgut was stained with anti-CSP antibody to visualize ruptured oocysts (green), and unruptured oocysts with parasite tdTomato signal (magenta) Scale bar: 100 µm. **b** Midguts from infected mosquitoes were dissected between days 14 to 20 post infection and ruptured and unruptured oocysts were visualized as shown in Panel A. For each midgut, the percent ruptured oocysts was calculated. Each dot represents one mosquito midgut and bars indicate the means.
