# Supplementary material for: Revisiting the Plasmodium sporozoite inoculum and elucidating the efficiency with which malaria parasites progress through the mosquito
Source: Nat Commun. 2024 Jan 25;15:748. doi: 10.1038/s41467-024-44962-4 (PMC10811227; doi:10.1038/s41467-024-44962-4)
Supplement: Supplementary file 3 — Reporting summary [file 41467_2024_44962_MOESM3_ESM.pdf]

nature portfolio

Corresponding author(s): Photini Sinnis, Sachie Kanatani

Last updated by author(s): Dec 22, 2023

Reporting Summary

Nature Portfolio wishes to improve the reproducibility of the work that we publish. This form provides structure for consistency and transparency in reporting. For further information on Nature Portfolio policies, see our Editorial Policies and the Editorial Policy Checklist.

Statistics

For all statistical analyses, confirm that the following items are present in the figure legend, table legend, main text, or Methods section.

n/a	Confirmed	
☐	☒	The exact sample size (*n*) for each experimental group/condition, given as a discrete number and unit of measurement
☐	☒	A statement on whether measurements were taken from distinct samples or whether the same sample was measured repeatedly
☐	☒	The statistical test(s) used AND whether they are one- or two-sided<br>*Only common tests should be described solely by name; describe more complex techniques in the Methods section.*
☐	☒	A description of all covariates tested
☐	☒	A description of any assumptions or corrections, such as tests of normality and adjustment for multiple comparisons
☐	☒	A full description of the statistical parameters including central tendency (e.g. means) or other basic estimates (e.g. regression coefficient) AND variation (e.g. standard deviation) or associated estimates of uncertainty (e.g. confidence intervals)
☐	☒	For null hypothesis testing, the test statistic (e.g. F, t, r) with confidence intervals, effect sizes, degrees of freedom and P value noted<br>*Give P values as exact values whenever suitable.*
☒	☐	For Bayesian analysis, information on the choice of priors and Markov chain Monte Carlo settings
☒	☐	For hierarchical and complex designs, identification of the appropriate level for tests and full reporting of outcomes
☐	☒	Estimates of effect sizes (e.g. Cohen's *d*, Pearson's *r*), indicating how they were calculated

Our web collection on statistics for biologists contains articles on many of the points above.

Software and code

Policy information about availability of computer code

Data collection	No custom software used.
Data analysis	Commercially availabe software, Prism (version 7 or 8.4) was used for data analysis. This information is included in the methods.

For manuscripts utilizing custom algorithms or software that are central to the research but not yet described in published literature, software must be made available to editors and reviewers. We strongly encourage code deposition in a community repository (e.g. GitHub). See the Nature Portfolio guidelines for submitting code & software for further information.

Data

Policy information about availability of data

All manuscripts must include a data availability statement. This statement should provide the following information, where applicable:

- Accession codes, unique identifiers, or web links for publicly available datasets
- A description of any restrictions on data availability
- For clinical datasets or third party data, please ensure that the statement adheres to our policy

All of the raw data used to make the main and supplementary figures are included in the Source Data excel file.

Research involving human participants, their data, or biological material

Policy information about studies with human participants or human data. See also policy information about sex, gender (identity/presentation), and sexual orientation and race, ethnicity and racism.

Reporting on sex and gender	N/A
Reporting on race, ethnicity, or other socially relevant groupings	N/A
Population characteristics	N/A
Recruitment	N/A
Ethics oversight	N/A

Note that full information on the approval of the study protocol must also be provided in the manuscript.

Field-specific reporting

Please select the one below that is the best fit for your research. If you are not sure, read the appropriate sections before making your selection.

☒ Life sciences ☐ Behavioural & social sciences ☐ Ecological, evolutionary & environmental sciences

For a reference copy of the document with all sections, see nature.com/documents/nr-reporting-summary-flat.pdf

Life sciences study design

All studies must disclose on these points even when the disclosure is negative.

Sample size	From our previous study on infection likelihood after a single P. yoelii mosquito bite (Aleshnick et al., PLoS Path 2020) we had determined that a minimum of 100 data points were needed to see a signal in what are inherently overdispersed data. Thus, for the two major findings of the paper, i.e. inoculum size and efficiency of salivary gland entry, we had greater than or equal to 100 data points.
Data exclusions	There were no data exclusions.
Replication	All experiments were performed with a minimum of 3 biological replicates. Occassionally there was a batch of mosquitoes that were not well infected and in these cases, we did not use the cage for experiments. We state in the Methods that we used cages with infection prevalence of >85%.
Randomization	Radomization was not applicable to our study - there were no separate experimental groups as we were investigating parasite numbers over the course of the lifecycle.
Blinding	Blinding was not applicable to our study because there were no separate control and experimental groups.

Reporting for specific materials, systems and methods

We require information from authors about some types of materials, experimental systems and methods used in many studies. Here, indicate whether each material, system or method listed is relevant to your study. If you are not sure if a list item applies to your research, read the appropriate section before selecting a response.

Materials & experimental systems

n/a	Involved in the study	
☐	☒	Antibodies
☒	☐	Eukaryotic cell lines
☒	☐	Palaeontology and archaeology
☐	☒	Animals and other organisms
☒	☐	Clinical data
☒	☐	Dual use research of concern
☒	☐	Plants

Methods

n/a	Involved in the study	
☒	☐	ChIP-seq
☒	☐	Flow cytometry
☒	☐	MRI-based neuroimaging

Antibodies

Antibodies used	mAb 2A10, mAb2F6, Alexa 488 goat anti-mouseIgG (Thermofisher #A11029)
Validation	mAb 2A10 is described in the following reference.<br>Nardin, E. et al. Circumsporozoite proteins of human malaria parasites Plasmodium falciparum and Plasmodium vivax. J. Exp. Med. 156, 20–30 (1982).<br>mAb2F6 is described in the following reference.<br>Sack, B. K. et al. Model for In Vivo Assessment of Humoral Protection against Malaria Sporozoite Challenge by Passive Transfer of Monoclonal Antibodies and Immune Serum. Infect. Immun. 82, 808–817 (2014).

Animals and other research organisms

Policy information about studies involving animals; ARRIVE guidelines recommended for reporting animal research, and Sex and Gender in Research

Laboratory animals	Swiss Webster (Taconic) 5-10 weeks old<br>Anopheles stephensi mosquitoes (Liston strain) 3-7 days post-emergence
Wild animals	We did not use wild animals
Reporting on sex	Though we used mice to generate infected mosquitoes it was the mosquitoes that were being studied. For these mosquito cycles we use outbred female mice as they are easier to work with and there are no data indicating that the sex of the mouse has any impact on the infection in the mosquito.<br>We also used mice for our probing experiments - infected mosquitoes were allowed to probe on the ear of mice which again were outbred female mice because of the ease of working with them. We would not anticipate that there would be a difference in mosquito behavior on male and female mouse ears. Furthermore our colleagues who performed similar experiments on artificial skin had very similar results (Andolina et al., In press eLife).
Field-collected samples	We did not use field-collected samples.
Ethics oversight	Johns Hopkins University Animal Care and Use Committee (Protocol #M020H267)

Note that full information on the approval of the study protocol must also be provided in the manuscript.

Plants

Seed stocks	N/A
Novel plant genotypes	N/A
Authentication	N/A
